# Flavonoids against the Warburg phenotype—concepts of predictive, preventive and personalised medicine to cut the Gordian knot of cancer cell metabolism

**DOI:** 10.1007/s13167-020-00217-y

**Published:** 2020-07-30

**Authors:** Marek Samec, Alena Liskova, Lenka Koklesova, Samson Mathews Samuel, Kevin Zhai, Constanze Buhrmann, Elizabeth Varghese, Mariam Abotaleb, Tawar Qaradakhi, Anthony Zulli, Martin Kello, Jan Mojzis, Pavol Zubor, Taeg Kyu Kwon, Mehdi Shakibaei, Dietrich Büsselberg, Gustavo R. Sarria, Olga Golubnitschaja, Peter Kubatka

**Affiliations:** 1 Clinic of Obstetrics and Gynecology, Jessenius Faculty of Medicine, Comenius University in Bratislava, 03601 Martin, Slovakia; 2 Department of Physiology and Biophysics, Weill Cornell Medicine in Qatar, Education City, Qatar Foundation, 24144, Doha, Qatar; 3 Musculoskeletal Research Group and Tumour Biology, Chair of Vegetative Anatomy, Institute of Anatomy, Faculty of Medicine, Ludwig-Maximilian-University Munich, 80336 Munich, Germany; 4 Institute for Health and Sport, Victoria University, Melbourne, VIC 3011 Australia; 5 Department of Pharmacology, Faculty of Medicine, P. J. Šafarik University, 040 11 Košice, Slovakia; 6 Department of Gynecologic Oncology, Norwegian Radium Hospital, Oslo University Hospital, 0379 Oslo, Norway; 7 OBGY Health & Care, Ltd., 01001 Zilina, Slovak Republic; 8 Department of Immunology and School of Medicine, Keimyung University, Dalseo-Gu, Daegu, 426 01 South Korea; 9 Department of Radiation Oncology, University Hospital Bonn, Rheinische Friedrich-Wilhelms-Universität Bonn, Bonn, Germany; 10 Predictive, Preventive Personalised (3P) Medicine, Department of Radiation Oncology, University Hospital Bonn, Rheinische Friedrich-Wilhelms-Universität Bonn, Bonn, Germany; 11 Department of Medical Biology, Jessenius Faculty of Medicine, Comenius University in Bratislava, 036 01 Martin, Slovakia

**Keywords:** Predictive preventive personalised medicine (PPPM / 3PM), Cancer, Warburg phenotype, Flavonoids, Anticancer effect, Cell metabolism, Co-morbidities, Malignancy, Disease manifestation, Age, Patient stratification, Aggressive metastatic disease, Multi-omics, Biomarker patterns, Liquid biopsy, Modifiable risk factors, Risk assessment, Microcirculation, Systemic hypoxia, Ischemic lesions, Prognosis, Individualised patient profiles, Treatment algorithms, Liver malignancy, Triple-negative breast cancer, Prostate cancer, Pregnancy, Chemoresistance, Radioresistance, Glucose metabolism, Oxidative phosphorylation, Proliferation, Metabolic reprogramming, Positron emission tomography, Magnetic resonance spectroscopy, Tumour imaging, FDG-PET, Glucose intake, PET-CT, Individual outcome, Palliative medicine, Polyphenols, Glycolysis, Carcinogenesis, Prognostic markers, Aerobic glycolysis, Glycolytic inhibitors, Pleiotropic activity, HIF-1

## Abstract

The Warburg effect is characterised by increased glucose uptake and lactate secretion in cancer cells resulting from metabolic transformation in tumour tissue. The corresponding molecular pathways switch from oxidative phosphorylation to aerobic glycolysis, due to changes in glucose degradation mechanisms known as the ‘Warburg reprogramming’ of cancer cells. Key glycolytic enzymes, glucose transporters and transcription factors involved in the Warburg transformation are frequently dysregulated during carcinogenesis considered as promising diagnostic and prognostic markers as well as treatment targets. Flavonoids are molecules with pleiotropic activities. The metabolism-regulating anticancer effects of flavonoids are broadly demonstrated in preclinical studies. Flavonoids modulate key pathways involved in the Warburg phenotype including but not limited to PKM2, HK2, GLUT1 and HIF-1. The corresponding molecular mechanisms and clinical relevance of ‘anti-Warburg’ effects of flavonoids are discussed in this review article. The most prominent examples are provided for the potential application of targeted ‘anti-Warburg’ measures in cancer management. Individualised profiling and patient stratification are presented as powerful tools for implementing targeted ‘anti-Warburg’ measures in the context of predictive, preventive and personalised medicine.

## Introduction

Despite significant progress in the therapy of oncological diseases in the twenty-first century, cancer remains a leading cause of death globally [1]. The association of neoplastic transformation with alterations at the metabolic level has been studied for almost a century. Malignant cells show profound alterations in their metabolic pathways changing the intra- and extracellular biochemistry that is associated with increased proliferation and angiogenesis [2]. Metabolic reprogramming leads to the switch from oxidative phosphorylation to aerobic glycolysis that represents a well-recognised signature of cancer energy metabolism associated with diminished drug-mediated apoptosis or chemo/radio-resistance of tumour cells [3, 4]. Like an orchestra, the enzymes that contribute to glycolysis represent individual players closely cooperating in the glucose degradation cascade. Specific differences in their enzymatic activities or expression rates act as prognostic biomarkers in cancer. The multi-omics (epigenomics, proteomics, transcriptomics, metabolomics) approach introduces novel challenges to the detection of individual signatures directly connected to cancer development as a consequence of metabolic disequilibrium of tumour cells [5, 6]. Acquired data from the multi-omic technologies is applicable in the novel clinical trend focused on predictive, preventive and personalised (3P) medicine strategies considered as the medicine of the future [5, 7]. During the last decade, increasing interest is recorded in targeting of essential steps of aerobic glycolysis as a promising approach to anticancer research [8]. To this end, several enzymatic inhibitors aimed at essential components of glucose metabolism have been tested in clinical trials. However, their application in routine clinical practice remains is not yet established [4, 9]. Increased aerobic glycolysis promotes the growth of various cancers; therefore, it is necessary to develop new therapeutic approaches to prevent metabolic reprogramming in cells [10]. Since ancient times, humans have been looking for therapeutic drugs against their diseases in nature [11]. Long-lasting investigations of medicinal plants led to the discoveries of numerous bioactive compounds that are now defined as phytochemicals [12]. Naturally occurring phytochemicals are promising agents against the initiation, promotion and progression of cancer with multiple mechanisms of action [13, 14]. Flavonoids, which represent a broad class of bioactive compounds in plants, are found in various functional foods. Recent evidence suggests beneficial functions of flavonoids in human health. The regular consumption of flavonoids is associated with the prevention of numerous chronic diseases including cancer [15]. Flavonoids inhibit carcinogenesis through multiple pathways [16, 17]. Amongst these, the modulation of cellular energy metabolism represents an extraordinarily interesting topic in the field of anticancer research. As mentioned above, there is a need to identify novel therapeutic approaches, and flavonoids could act as promising metabolism-regulating agents in suppressing malignant transformations of cells.

### Aim of the study

This paper focuses on the anticancer potential of flavonoids—specifically, those related to the suppression of the Warburg effect in cancer cells. Firstly, this review discusses fundamental aspects of the Warburg effect, glycolytic enzymes and molecular pathways directly regulating cancer energy metabolism. The core of our article summarises experimental studies evaluating the anticancer effects of flavonoids via modulation of essential steps and compartments of glycolysis. The beneficial role of flavonoids in cancer metabolism is well-documented in preclinical research; in this review, we emphasise the need for targeted clinical cancer research on the effects of flavonoids in cellular metabolic reprograming.

### Data sources

Data were obtained from the English-language biomedicine literature by the use of ‘flavonoids’ or ‘polyphenols’ or ‘cancer’ or ‘Warburg effect’ and ‘glycolysis’ as either a keyword or medical subject heading (MeSH) term in searches of the PubMed database (years 2015–2020).

## Fundamental aspects of cancer metabolism

**Fig. 1:**
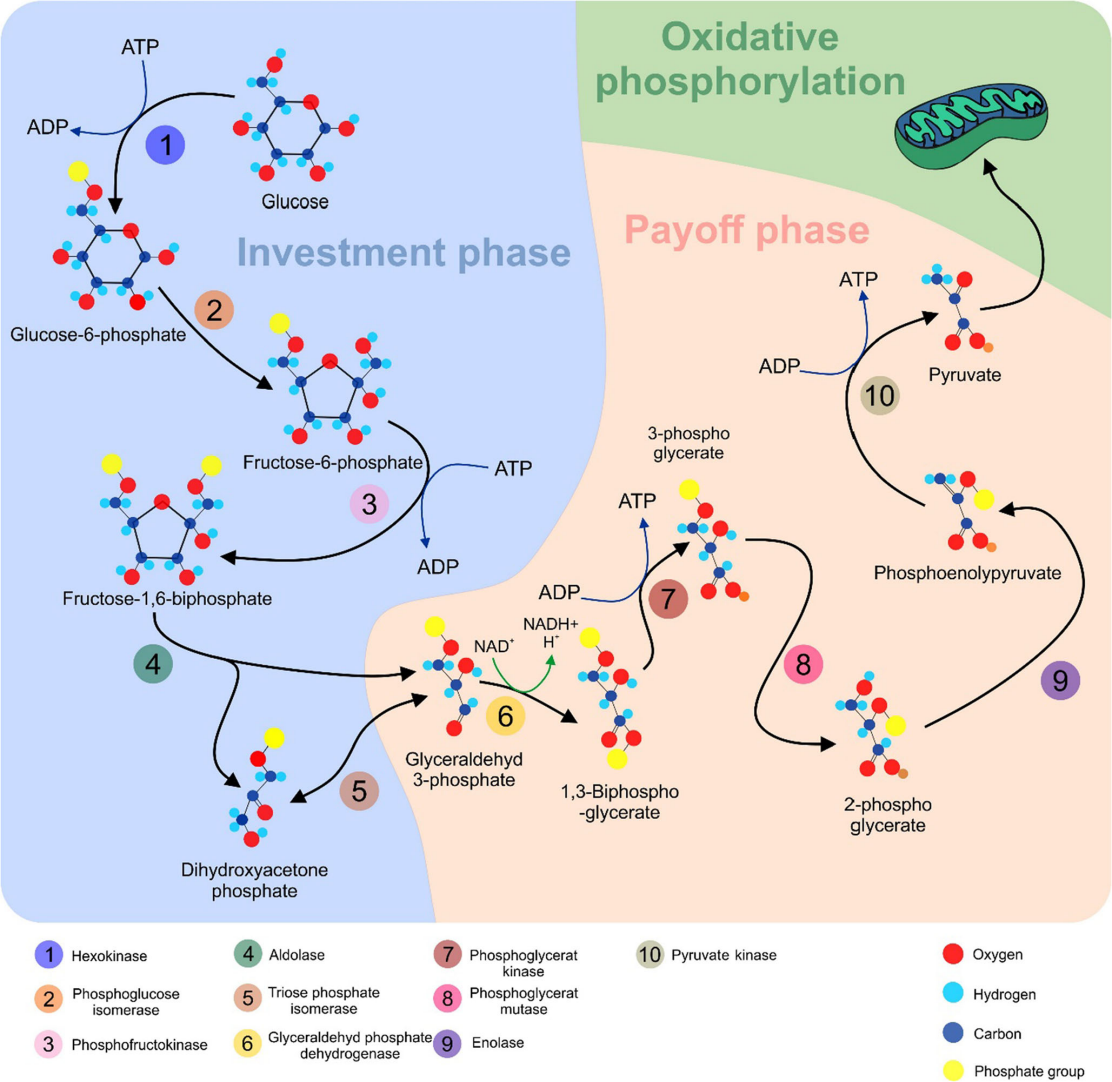
Metabolism of glucose. The investment phase begins with the first enzyme, hexokinase (HK), which adds a phosphate group to glucose leading to the generation of glucose-6-phosphate (G6P). In the next step, phosphoglucose isomerase (PGI) modifies G6P into fructose-6-phosphate (F6P). The enzyme phosphofructokinase (PFK1) transforms F6P into fructose-1,6-bisphosphate (FBP). FBP is further lysed into dihydroxyacetone (DHAP) and glyceraldehyde 3-phosphate (G3P) by fructose-bisphosphate aldolase (aldolase). The enzyme triosephosphate isomerase (TPI) converts DHAP into G3P. The payoff phase begins with the metabolisation of G3P into 1,3-bisphosphoglycerate (1,3BGP) by glyceraldehyde-3-phosphate dehydrogenase (GAPDH). Next, phosphoglycerate kinase (PGK) converts 1,3BGP into 3-phosphoglycerate (3PG). Phosphoglycerate mutase (PGM) generates 2-phosphoglycerate (2PG) from the 3PG, and 2PG then converts enolase into phosphoenolpyruvate (PEP). Pyruvate kinase (PK) catalyses the final step, in which PEP is transformed into pyruvate [19, 20]. Subsequently, pyruvate undergoes oxidative phosphorylation in mitochondria resulting in the generation of 36 molecules of ATP from the tricarboxylic acid cycle (TCA). ATP, adenosine triphosphate; ADP, adenosine diphosphate; NAD^+^, nicotinamide adenine dinucleotide (oxidised form); NADH, nicotinamide adenine dinucleotide (reduced form)

The cross-connection between cancer and altered metabolism is well characterised in cancer research. Almost 100 years ago, Otto Heinrich Warburg and colleagues observed that molecules of glucose undergo predominantly aerobic glycolysis in tumours, even under normoxic conditions, to support rapid cell division. In contrast, normal cells primarily utilise oxidative phosphorylation to generate adenosine triphosphate (ATP) in the presence of oxygen. The observation mentioned above became known as ‘the Warburg effect’ [18]. Glucose metabolism represents an enzymatic cascade consisting of two phases: the investment phase and the payoff phase, which involve several enzymes (Fig. 1).

The above pathway illustrates the metabolism of glucose under aerobic conditions in normal cells (Fig. 1). Conversely, in cancer cells, pyruvate is transformed into lactate by lactate dehydrogenase A (LDHA) and the yield of ATP is far lower, as only two ATP molecules are released per molecule of glucose through glycolysis [21, 22]. Although glycolysis is less efficient than oxidative phosphorylation in generating ATP, it produces energy more rapidly per unit of glucose [23]. An initial hypothesis predicted mitochondrial defects in cancer cells (i.e. lack of oxidative phosphorylation) and, thus, postulated aerobic glycolysis as the necessary way to produce ATP [24]. However, the rate of glucose uptake in cancer cells is dramatically elevated and lactate is created even in the presence of functional mitochondria [25]. Moreover, defective mitochondria are rather rare, and tumour cells maintain the ability of oxidative phosphorylation [26]. Proliferating cells are constantly supplied with glucose and various nutrients. Additionally, these cells require high ATP/ADP, NAD+/NADH and NADP+/NADPH ratios [25, 27]. Any imbalance between these molecules leads to the suppression of proliferation. Therefore, oxidative phosphorylation is not suitable for the maintenance of the above-mentioned ratios; however, for biosynthetic purposes, it produces ATP, while NAD+/NADH and NADP+/NADPH are under-dimensioned [28]. The preference of aerobic glycolysis to oxidative phosphorylation in cancer cells is linked to external conditions such as low extracellular pH and hypoxia [29]. Moreover, the abnormalities in signalling pathways as consequences of certain genetic aberrations promote cancer-associated glycolysis [30]. Thus, the determination of the cancer metabolism phenotype characterised by increased glycolytic activity is controlled by intrinsic alterations as well as external responses of the cell to the tumour microenvironment [31].

## Molecular view into the Warburg effect

Recent advances in the fields of genetics and molecular biology can shed light on the principles and mechanisms of aerobic glycolysis in tumours. Disequilibrium in the PI3K signalling pathway is frequently detected in various cancers [32–35]. These aberrations of PI3K support the biological processes such as proliferation and invasion via modification of glucose metabolism. Evidence suggests that mutation in PIK3CA promotes glycolysis in cervical cancer cells [36]. Akt1, the downstream effector of PI3K, is a major player in cancer. Akt1 stimulates various intracellular events associated with elevated levels of glycolysis including translocation, stability and overexpression of glucose transporters (GLUT1, 2 and 4) [37–39], or negatively via suppression of forkhead box subfamily O1 (FOXO1) acting as a repressor of glycolysis. Akt1 activates mTOR which plays a crucial role in protein synthesis and cell proliferation. Moreover, mTOR is essential for glucose uptake, lipid biosynthesis and glycolysis [40]. In addition, mTOR induces hypoxia-inducible factor-1 (HIF-1) activity, even under normoxic conditions resulting in aerobic glycolysis [41]. HIF-1 can also be activated by the loss of von Hippel–Lindau (pVHL) regulation either as a direct or indirect consequence of mutations of succinate dehydrogenase (SDH) or fumarate hydratase (FH) [42]. The transcription factor c-Myc is an oncogene modulating various intracellular processes, including proliferation and growth. In addition, it has been suggested that c-Myc and HIF-1 cooperate in the regulation of glycolysis [43]. Oncoprotein c-Myc is associated with increased expression of GLUT genes [44] or enzymes such as LDHA [45] and PDK1 [46]. In contrast to Akt1, which is connected to enhanced proliferation and aerobic glycolysis via activation of mTOR, AMP-activated kinase (AMPK) suppresses mTOR signalling [47] that depends on the AMP/ATP ratio in the cell. When the AMP/ATP ratio is low, AMPK phosphorylates enzymes responsible for the generation of ATP via oxidative phosphorylation [48]. Activation of AMPK is mediated via liver kinase B1 (LBK1) which supports the maintenance of an intracellular energy balance and negatively regulates the Warburg effect. Dysfunction of the LBK1/AMPK pathway is defined as a hallmark of cancer progression [49].

P53 is a well-known tumour suppressor with a crucial role in stress stimuli responses and in the initiation of cell cycle arrest, senescence or apoptosis [9, 36]. Importantly, p53 negatively regulates aerobic glycolysis by promoting TP53-induced glycolysis and apoptosis regulator (TIGAR) affecting glycolysis enzymes and synthesis of cytochrome oxidase 2 (SCO2) which participates in mitochondrial metabolism under normoxia [50, 51]. Recent evidence suggests that the transcription factor sine oculis homeobox 1 (SIX 1) contributes to the control of many glycolytic enzymes associated with tumour metabolism [52]. The transcription factor OCT1 is frequently upregulated in cancer. Recent experimental studies have demonstrated its role in the silencing of oxidative phosphorylation and switching to aerobic glycolysis [53]. Additionally, an oncogenic transcription factor, Yin Yang 1 (YY1), promotes aerobic glycolysis via upregulation of GLUT3 [54].

Pyruvate kinase plays an essential role in the transformation of normal glucose metabolism into the aerobic glycolytic phenotype responsible for transforming PEP into pyruvate and production of ATP. In mammals, four isoforms of pyruvate kinase, namely PKL, PKR, PKM1 and PKM2, exist [55]. The PKM1 isoform has greater enzymatic efficiency than the PKM2 form. Paradoxically, the less efficient M2 form is preferred in tumours due to its promotion of c-Myc [56]. Oncoprotein c-Myc affects the splicing of PK mRNA by upregulating polypyrimidine tract binding protein (PTB) and heterogeneous nuclear ribonucleoproteins (hnRNPs) A1 and A2 leading to the predominant production of PKM2 [57]. Less efficient PKM2 is advantageous for cell proliferation as it allows for entering carbohydrate metabolites of glycolysis into alternative pathways to produce the macromolecules and NADPH necessary for tumour growth [58].

### Epigenetic regulation of the Warburg effect

Based on current research, epigenetic alterations such as methylation of DNA, histone modifications or miRNA expression manifest tight cross-connections that modulate cancer metabolism. All enzymes and proteins that participate in glycolysis can be post-transcriptionally regulated by miRNAs. Glucose transporters, as mentioned above, are associated with glucose uptake in cells and their upregulation is a hallmark of the Warburg phenotype in cancer cells. Many experimental studies suggest an association between miRNA deregulation and GLUT1 activity including upregulation of miR-150 [59], miR-522-3p [60] and miR-10a [61], whereas expression rates of miR-340 [62], miR-218 [63] and miR-200c [64] are reduced.

The glycolytic enzyme HK2 is upregulated in cancer. MiR-143 has been shown to repress HK2 in colon cancer cells [65]. On the other hand, overexpression of miR-155 is correlated with the upregulation of HK2 in lung cancer cells [66]. PKM2 is also overexpressed in cancer metabolism. Current evidence indicates tumour-suppressive functions of miR-148a and miR-326 in the downregulation of PKM2 in thyroid cancer cells [67]. In addition, miRNA-let-7a downregulates PKM2 in cervical cancer cells [68]. Expression of small non-coding RNAs miR-1 and miRNA-133b can suppress the expression of PKM2 via interaction with PKTB1 and can also regulate the splicing of PKM2 [69]. Interestingly, miRNAs control cancer metabolism indirectly via regulation of long non-coding RNA. MiR-586 can form a competing endogenous RNA model with LncRNA-MIF and thus suppress glycolysis via degradation of c-Myc by Fbcw7 (E3 ligase for c-Myc) [70].

Methylation, another regulatory mechanism affecting gene expression, is also involved in the Warburg effect. Intragenic methylation mediated by binding of the protein BORIS leads to an alternative splicing of PKM resulting in the predominant generation of PKM2 and consequent development of cancer-related glycolysis [71]. Furthermore, overexpression of HK2 mediated by hypomethylation occurs in glioblastoma multiforme [72].

**Fig. 2:**
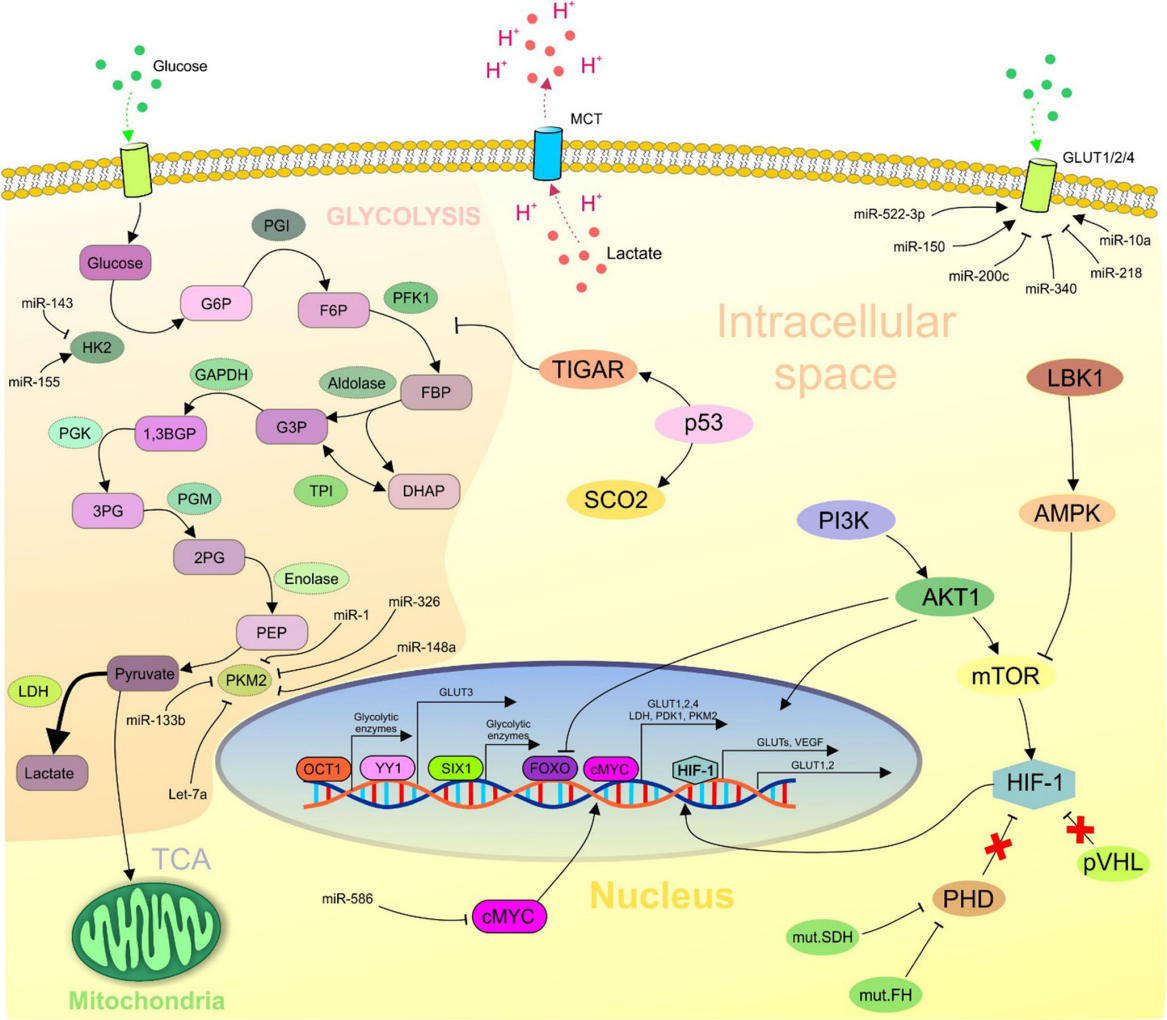
Molecular mechanisms contributing to the Warburg effect in cancer cells. 6GP, glucose-6-phosphate; 6FP, fructose-6-phosphate; FBP, fructose-1,6-bisphosphate; DHAP, dihydroxyacetone; G3P, glyceraldehyde 3-phosphate; 1,3BGP, 1,3-bisphosphoglycerate; 3PG, 3-phosphoglycerate; 2PG, 2-phosphoglycerate; PEP, phosphoenolpyruvate; HK2, hexokinase 2; PGI, phosphoglucose isomerase; PFK1, phosphofructokinase; TPI, triosephosphate isomerase; GAPDH, glyceraldehyde-3-phosphate dehydrogenase; PGK, phosphoglycerate kinase; PGM, phosphoglycerate mutase; PKM2, pyruvate kinase 2; LDH, lactate dehydrogenase; TCA, tricarboxylic acid cycle

Likewise, epigenetic changes regulated by histone modifications also participate in the interplay between metabolism and cancer. Overproduction of lactate is a fundamental characteristic of the transformation of pyruvate into lactate. An interesting way of how lactate promotes cancer phenotypes in non-metabolic roles is demonstrated by lactylation of histones in macrophage leading to the expression of genes such as *Arg1*, which are involved in the M2-like polarisation of tumour-associated macrophages [73]. Moreover, the monoubiquitinating of histone H2B (H2Bub1) negatively regulates the Warburg effect. Recent evidence shows a cross-connection between elevated levels of PKM2 and decreases in H2Bub1 that indicate the oncogenic function of PKM2 via its control of histone modifications [74]. Figure 2 summarises the molecular mechanisms characteristic for cancer metabolism.

### Tumour microenvironment in the regulation of aerobic glycolysis

In contrast to genetic alterations associated with the transformation of metabolic pathways and consequent aerobic glycolysis, the tumourigenic microenvironment of the Warburg phenotype gives selective advantages in the growth and proliferation of tumour cells via several distinct mechanisms. Elevated glucose intake and consequent promotion of glucose metabolism increase the acidity of the microenvironment. Accumulation of lactate as the final product of aerobic glycolysis and the need of neutral pH for cell maintenance lead to its secretion via monocarboxylate transporters (MCT) together with H^+^ ions into extracellular space [75]. Increased microenvironmental acidity has multiple benefits for tumour cell proliferation and invasion [76]. Extracellular lactate activates growth factors including vascular endothelial growth factor (VEGF), tumour growth factor β (TGF β) and cytokine interleukin 1 (IL-1) [77]. Moreover, acidic tumour microenvironment promotes the activation of proteinases such as matrix metalloproteinase 9 (MMP-9) and enhances the invasiveness of cancer [78]. Low pH in the tumour region affects immune cells and immune responses [79]. Lactic acid, secreted by cancer cells, contributes to M2-macrophage polarisation [80]. Tumour-derived lactate is an intrinsic inflammatory factor associated with the promotion of chronic inflammatory responses through IL-17, which is secreted by T cells and macrophages [81, 82]. Although extracellular lactate does not modify the differentiation of monocytes into dendritic cells (DCs), it alters the antigen presentation process mediated by DCs and regulates the optimal functionality of DCs [83].

Despite progress in cancer-related research, multiple challenges including the repression of aerobic glycolysis in carcinogenesis still exist. Up to now, several glycolytic inhibitors, which contribute to the blockade of essential enzymes in glucose metabolism, have been tested against tumours in preclinical and clinical trials (Table 1).

**Table 1:** Selected inhibitors of glycolysis

Target	Agent	Cancer	References
PKM2	TLN-232/CAP-232	Renal, melanoma	[84, 85]
HK2	2-Deoxyglucose	Prostate	[86, 87]
	3-Bromopyruvate	Colorectal, breast, hepatocellular	[84, 88, 89]
LDHA	FX 11	Lymphoma, pancreatic	[90]
	Oxamate	Nasopharyngeal	[91]
PDH	CPI-613	Small cell lung, pancreatic	[92, 93]
PDK	Dichloroacetate	Breast	[94, 95]
PFKFB3	3PO	Bladder, tongue	[96, 97]
GLUT1/3	STF-31	Renal	[98]

*PKM2*, pyruvate kinase muscle isoform 2; *HK2*, hexokinase 2; *LDHA*, lactate dehydrogenase A; *PDH*, pyruvate dehydrogenase; *PDK*, pyruvate dehydrogenase kinase; *PFKFB3*, 6-phosphofructo-2-kinase/fructose-2,6-bisphosphatase 3; *GLUT1/3*, glucose transporter 1/3

## Flavonoids regulating glucose metabolism

**Fig. 3:**
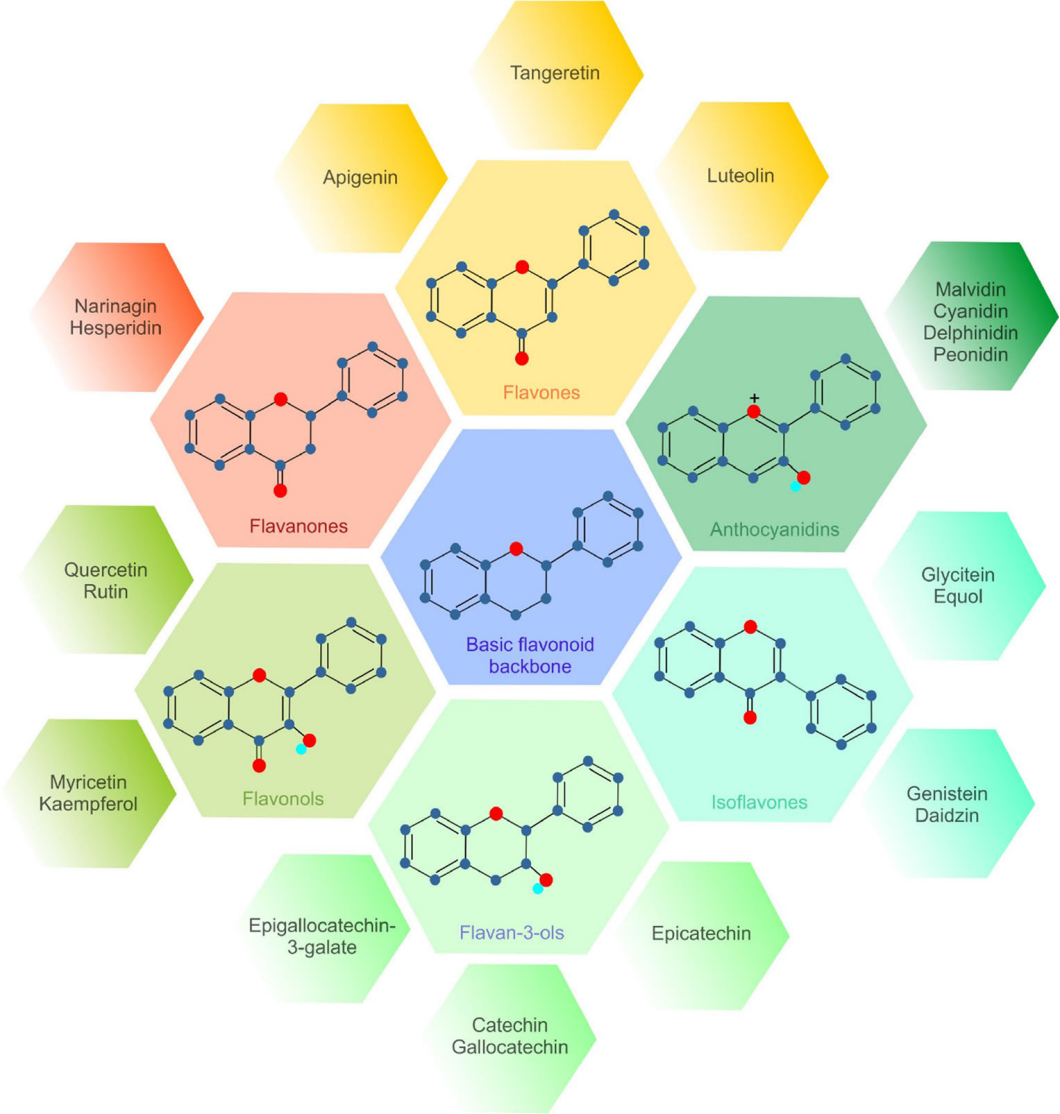
Structures of the main subgroups of flavonoids

Plant-derived secondary metabolites, known as phytochemicals, exert numerous beneficial effects on human health. Many pre/clinical studies have suggested their anticancer functions at the epigenetic and proteomic levels of action [99–105]. The broad spectrum of salubrious properties and the enormous diversity within the classification predetermine phytonutrients for further analysis of their role in the regulation of tumourigenesis. Flavonoids represent a diverse group of phytochemicals (Fig. 3) that exhibit antioxidative, antiangiogenic and overall antineoplastic efficacy [14, 106]. Switching from oxidative phosphorylation to aerobic glycolysis, even under normoxic conditions, is associated with cancer transformation [107]. There is increasing evidence for the importance of flavonoids in modulating carcinogenic pathways associated with glucose metabolism. Flavonoids target the regulation of the activity of certain enzymes involved in aerobic glycolysis, expression of transporters responsible for glucose uptake, modulation of HIF-1 under normoxic conditions and many other parameters within the Warburg phenotype. Flavonoids thus represent a potential therapeutic approach for oncology-related research.

### PKM2

As described above, PKM2 supports metabolic alterations associated with the switch from benign phenotype to the malignant one [108]. Naturally occurring flavonoids are suggested as efficient regulators of aerobic glycolysis via modulation of PKM2 activity. Apigenin (AP), a common dietary flavonoid found in vegetables, exerts various benefits, including anti-inflammatory, antiviral and antioxidant effects [109]. Moreover, AP is an anticancer agent inhibiting several molecular pathways of tumour development [110]. Regarding glucose metabolism, this secondary metabolite blocked glycolysis through regulation of PKM2 activity and expression in a colon cancer cell line (HCT116). Additionally, AP is regarded as a potential allosteric inhibitor of PKM2. AP could maintain a low PKM2/PKM1 ratio as a consequence of inhibition of the β-catenin/c-Myc/PTBP1 pathway [111]. Similarly, proanthocyanidin B2 (PB2) affected the activity of PKM2 in several hepatocellular cancer cell lines (HCC-LM3, Bel-7402, SMMC-7721, Huh-7 and HepG2). Experimental data demonstrated decreased levels of PKM2 mediated by PB2 via an inhibition of nuclear translocation and expression of the analysed enzyme due to the disruption of an interaction between PKM/HSP90/Hif-1α in HCC cells [112]. Additionally, epigallocatechin-3-gallate (EGCG), a flavonoid found in green tea, demonstrated anticancer activity in many experimental studies [113–116]. Wei et al. tested the effects of EGCG (concentrations of 2, 4 and 8 μM) in a breast cancer cell line (4T1) focusing on glucose metabolism in vitro. At its highest concentration, EGCG significantly decreased the enzymatic activity and mRNA level of pyruvate kinase [117].

The inhibitory effects of several polyphenols on cancer were analysed by an evaluation of PKM2 enzymatic activity in vitro*.* Amongst these polyphenols, (±)-taxifolin (TAX), neoeriocitrin (NEO), fisetin (FIS), (−)-catechin gallate (CG) and (−)-epicatechin (EP) exerted the greatest inhibitory efficacy on PKM2 activity [118]. Moreover, quercetin (QUE), a plant flavonol, significantly decreased the level of glycolysis-related proteins including PKM2. Similarly, western blotting documented a decrease in PKM2 level through modulation of the Akt–mTOR pathway in vivo [119]. Additionally, QUE reduced the level of PKM2 in the colon mucosa of F344 rats in the study pointing to a chemopreventive role of the flavonoid [120]. Shikonin (SHI) is a natural flavonoid occurring in *Lithospermum erythrorhizon*. Chen et al. investigated the anticancer activity of SHI and its analogue alkannin against drug-sensitive and resistant cancer cells (MCF-7, MCF-7/Adr, MCF-7/Bcl-xL, MCF-7/Bcl-2 and A549) via PKM2 suppression and consequent inhibition of glycolysis. The results showed that both tested compounds repressed PKM2 activity without affecting other PKM isoforms [121]. Finally, SHI suppressed tumour growth in mice with implanted B16 melanoma cells by reducing PKM2-mediated aerobic glycolysis [122].

### HK2

Hexokinase (HK) is the enzyme responsible for the phosphorylation of hexoses, which is the first irreversible step of glycolysis [123]. HK (primarily HK2) is often overexpressed in many types of cancer and may represent a potential molecular target for therapy using flavonoids [124–127]. Xanthohumol (XA), a flavonoid isolated from *Humulus lupulus*, suppressed the growth and proliferation of many tumours in preclinical studies [128–131]. Recently, a study evaluated the impact of XA on colorectal cancer progression via the downregulation of HK2-mediated glycolysis. XA inhibited HK2 and glycolysis through the suppression of EGFR–Akt signalling colon cancer cell lines (HCT116, HT29, SW620) in vitro and in a murine xenograft model in vivo [132]*.* As was demonstrated earlier, QUE exerted many anticancer effects in vivo and in vitro, even in the regulation of glucose metabolism, which leads to neoplastic transformation [133]. QUE reduced the level of HK2 and suppressed Akt/mTOR signalling in hepatocellular cancer lines (SMMG-7721, BEL-7402) in vitro. Additionally, QUE significantly decreased the level of HK2 and tumour growth in vivo [134]*.* Moreover, EGCG inhibited several essential enzymes that contribute to cancer metabolism. Amongst them, the enzymatic activity as well as the protein level of HK2 was significantly reduced in 4T1 cell line [117].

In addition to natural flavonoids and their roles in the chemopreventive and therapeutic aspects of cancer research, synthetic metabolites with broad anticancer properties have been reported [135]. The metabolic impact and biological activity of a set of new 3′,4′,5′-trimethoxy flavonoid salicylate derivatives were observed in the colorectal cancer cell line (HCT116). A synthetic compound, 10v, demonstrated cytotoxic potency against cancer cells. Results from western blotting suggested that 10v could inhibit cancer transformation and progression via downregulation of HK2 [136]. Another synthetic flavonoid, GL-V9, was tested against breast cancer cell lines (MCF-7 and MDA-MB-231). GL-V9 inhibited the expression of HK2 in both cell lines. Moreover, it promoted the dissociation of HK2 from a voltage-dependent anion-selective channel (VDAC) in the mitochondrial outer membrane resulting in suppression of the glycolytic pathway and consequent mitochondria-mediated apoptosis [137]. Additionally, Zhou et al. studied the cytotoxic activity of the synthetic flavonoid FV-429 against MDA-MB-231 cells. In a similar fashion as GL-V9, FV-429 triggered the apoptosis and inhibited glycolysis via dissociation of HK2 from VDAC in the mitochondrial membrane [138]. Moreover, the antineoplastic effects of the synthetic flavonoid Gen-27, mediated by the suppression of HK2, were observed in the MDA-MB-231 cell line. Application of Gen-27 to breast cancer cells led to a decrease in HK2 expression and the dissociation of HK2 from VDAC [139].

The antitumour and metabolic effects of phytochemicals are also mediated by the modulation of epigenetic machinery such as miRNAs. An individual miRNA can regulate the expression of multiple genes directly linked to glycolysis. Therefore, miRNAs are possible targets for cancer prevention and therapy. The flavonoid astralangin (ASG) occurs in many traditional medicinal plants. ASG inhibited glycolysis and proliferation in hepatocellular cancer in vitro and in vivo. Experimentally, ASG reduced the expression of HK2 by upregulating miR-125b in HepG2 HCC and Huh-7 cells. In addition, in vivo experiment using Huh-7 xenografts in nude mice and H22 cells transplanted in Kunming mice demonstrated a tumour-suppressive effect of AGS. Tumour growth reduction was associated with higher expression of miR-125b which correlated with decreasing HK2 expression observed in tumour tissue [140].

### Additional glycolytic enzymes

Glycolysis, as the main metabolic cascade contributing to carbohydrate degradation, involves numerous enzymes with specific roles in the metabolic pathway [141]. Alterations of the essential enzymes PKM2 and HK2 participate in the Warburg phenotype of cancer cells. Aerobic glycolysis is a complex cascade involving other enzymes; specific changes in their enzymatic activity and/or expression rate promote tumourigenesis [142]. Naturally occurring flavonoids can suppress these changes. LDH catalyses the conversion of pyruvate into lactate. An elevated level of LDH is a biomarker of poor prognosis in cancer [143]. One possible means of cancer suppression is the application of synthetic or natural inhibitors. Morin (MO), a flavonoid isolated from the *Moraceae* family, inhibited the enzymatic activity of LDH. MO modulated the conformation and inhibited the catalytic activity of LDH [144]. Furthermore, QUE effectively suppressed cancer growth and proliferation in MCF-7 and MDA-MB-231 cells and decreased the level of glycolysis-related enzymes including LDHA [119]. Additionally, luteolin-7-O-β-D-glucoside (LU-7OβD), a flavonoid from *Phlomis curdica*, inhibited an isoform of human LDH. This property could be applied to improve anticancer treatment via the inhibition of the key glycolytic enzyme by luteolin [145]. Application of the polyphenol EGCG reduced the activity of LDH and PFK in 4T1 cells [117]. Furthermore, rats fed with QUE (10 g/kg diet for 11 weeks) exhibited enhanced cellular mechanisms involved in the prevention of carcinogenesis, while a transcriptome analysis showed downregulation of the oncogenic mitogen-activated protein kinase (MAPK). QUE also downregulated glycolytic enzymes including aldolase, GAPDH and α-enolase [120].

### GLUTs

Glucose transporters are essential proteins that regulate glycolytic flux in the cell. Many oncogenic pathways are connected to the regulation of GLUT [146]. Flavonoids can modulate these pathways thus influencing glucose metabolism in tumours. Genistein (GE), phloretin (PH), daidzein and AP were tested against prostate cancer androgen-sensitive (LNCaP) and androgen-insensitive (PC-3) cell lines. Glucose intake was measured using nonradiolabeled 2-deoxyglucose. GLUT1 and GLUT4 levels were evaluated by western blot and immunocytochemistry. Amongst the four flavonoids, AP and PH were the most efficient in regulating the level of GLUTs and glucose intake in prostate cancer cells [147]. Furthermore, AP has also been found to reduce glucose transport by downregulating GLUT1 at the mRNA and protein levels in the pancreatic cancer cell lines CD18 and S2-013 [148]. Hesperetin (HES) is a citrus flavonoid reported to reduce cholesterol in plasma [149, 150]. Yang et al. observed a metabolic impact of HES on breast cancer cells MDA-MB-231. Their data indicated a decrease in glucose intake due to GLUT1 downregulation. Moreover, HES reduced insulin-stimulated glucose uptake by impairing the cell membrane translocation of GLUT4 [151]. Kaempferol (KAE; tetrahydroxy flavone) is found in various plants and exerts wide anticancer properties [152]. Azavedo et al. studied alterations in the uptake of ^3^H-deoxy-d-glucose, a glucose analogue, in MCF-7 cells after treatment with myricetin, chrysin, KAE, resveratrol, genistein and XA. Amongst these flavonoids, KAE was found to be the most effective inhibitor of glucose analogue uptake. Long-term KAE exposure also inhibited the ^3^H-deoxy-d-glucose uptake via downregulation of GLUT1 mRNA level in MCF-7 cells [153]. Another study utilised ^3^H-deoxy-d-glucose to estimate a glucose uptake in MCF-7 and MDA-MB-231 cells treated with QUE and EGCG. Both polyphenols affected glucose intake via direct and competitive inhibition of GLUT1 and thus suppressed the glucose metabolism [154]. In addition, EGCG demonstrated anticancer efficacy against 4T1 via reduction of GLUT1 expression [117]. Furthermore, QUE blocked an intake of glucose in MCF-7 and MDA-MB-231 through inhibition of GLUT1 activity as a consequence of the regulatory effect of QUE on the protein level of the glucose transporter [119]. Silibinin (SI), a naturally occurring flavone, is a major component of silymarin isolated from *Sylibum marianum*. SI inhibited GLUT4 in non-tumour CHO cells. Therefore, modulation of glucose transport via SI is a potential chemopreventive approach that could be applied also in cancer cells [155].

### HIF-1

**Fig. 4:**
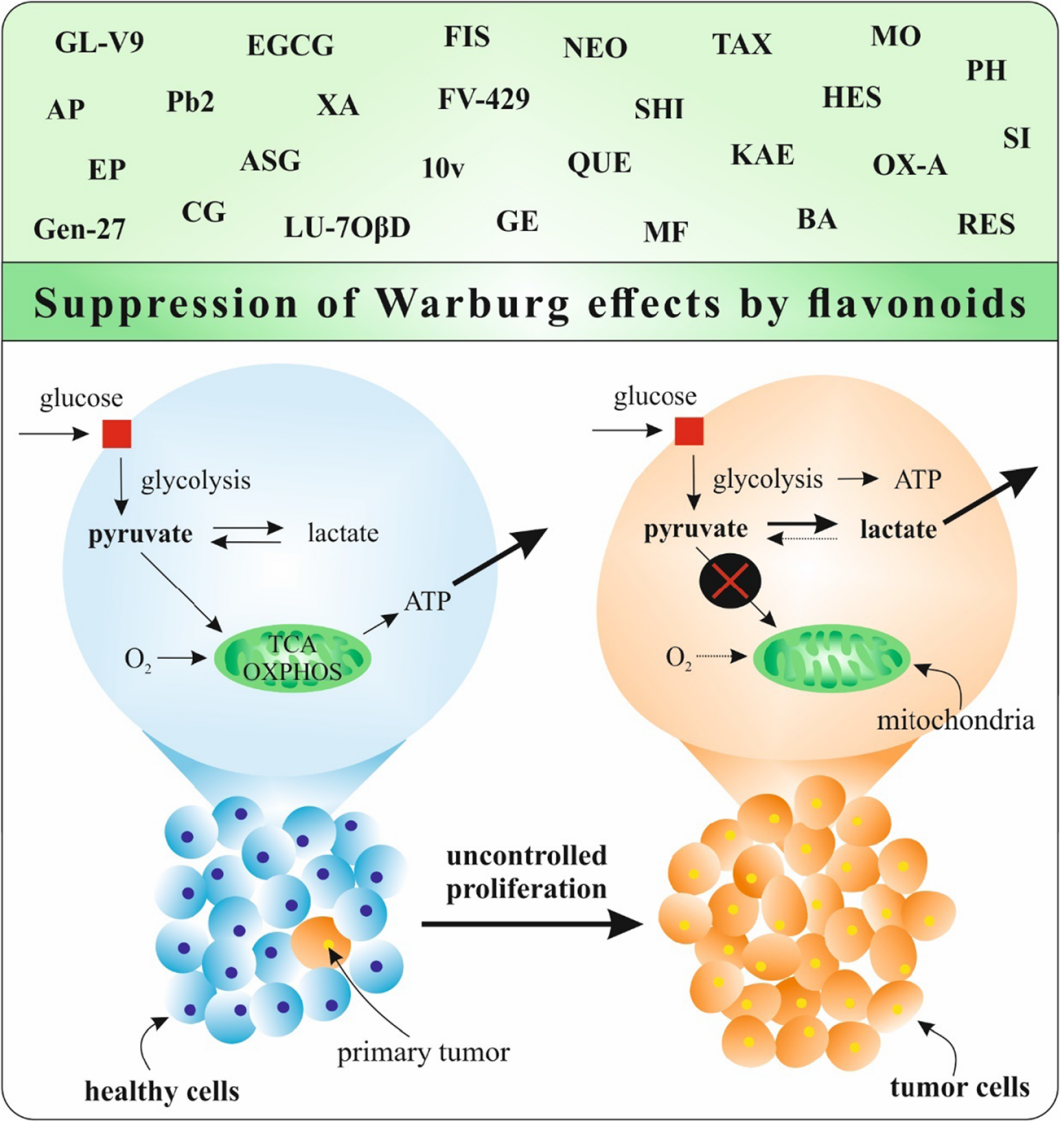
The role of flavonoids in the regulation of glucose metabolism in cancer. AP, apigenin; PB2, proanthocyanidin B2; EGCG, epigallocatechin-3-gallate; TAX, taxifolin; NEO, neoeriocitrin; FIS, fisetin; CG, catechin gallate; EP, epicatechin; QUE, quercetin; SHI, shikonin; XA, xantohumol; ASG, astralangin; MO, morin; LU-7OβD, luteolin-7-O-β-D-glucoside; PH, phloretin; HES, hesperitin; KAE, kaempferol; SI, silibinin; BA, baicalein; MF, methylalpinumisoflavon; OX-A, oroxylin A; RES, resveratrol; OXPHOS, oxidative phosphorylation; TCA, tricarboxylic acid cycle; ATP, adenosine triphosphate

HIF-1 is a transcription factor associated with the regulation of many pivotal pathways in healthy cells, and its alterations caused by genetic, epigenetic or intra/extracellular stimulation lead to cell transformation into the cancer phenotype. Elevated expression of HIF-1 promotes tumour-associated angiogenesis, proliferation and progression through the modulation of glycolytic cascades [156]. Current evidence suggests that flavonoids may play a considerable role in cancer treatment and have multiple potential targets in tumourigenesis, including HIF-1 [157]. Baicalein (BA; 5,6,7-trihydroxyflavone), a flavonoid isolated from *Scutellaria baicalensis*, exerts strong anticancer activity on various tumours [158–160]. A recent study evaluated the impact of BA on glycolysis-related chemoresistance to 5-fluorouracil in gastric cancer cells (AGS). BA increased the sensitivity of AGS cells to 5-fluorouracil therapy. The administration of this flavonoid led to the inhibition of hypoxia-induced Akt phosphorylation as a consequence of the improvement of PTEN accumulation associated with decreasing HIF-1α expression. Since BA suppressed glycolysis via PTEN/Akt/HIF-1α, it is a possible therapeutic sensitiser against gastric cancer [161]. Interestingly, other studies confirmed the inhibitory effects of flavonoids on HIF-1 in the regulation of glucose metabolism. For instance, methylalpinumisoflavon (MF), a flavonoid isolated from *Lonchocarpus glabrescens*, contributed to the regulation of glycolysis in vitro. MF demonstrated strong cytotoxic effects on T47D cells and also suppressed HIF-1 activation and its target genes including *CDKN1A*, *VEGF* and *GLUT-1* in cancer cells [162]. Moreover, oroxylin A (OX-A), a bioactive compound occurring in traditional Chinese medicinal plants, was tested against MDA-MB-231 cells. OX-A administration correlated with the inhibition of cancer-related glycolysis via Sirtuin-3 mediated destabilisation of HIF-1α controlling expression of glucose degradation enzymes [163]. Furthermore, EGCG reduced the expression of HIF-1α and enzymes related to glycolysis in T47D cells [117]. Interestingly, resveratrol (RES) reduced glucose uptake and glycolysis in the Lewis lung carcinoma (LLC), breast T47D and colon HT-29 cancer cell lines. Measurements of cellular uptake of the glucose analogue 18F-fluorodeoxyglucose (18F-FDG) during RES exposure indicated that RES repressed intracellular reactive oxidative species (ROS) and thereby decreased HIF-1α accumulation, reduced GLUT-1 expression and caused glycolytic flux [164]. Table 2 summarises the results of experimental studies investigating the effects of flavonoids on the critical components of glycolysis. Figure 4 is an overview of the flavonoids used as repressors of cancer-related metabolism.

**Table 2:** Flavonoids in the Warburg phenotype

Component of glycolysis	Flavonoid	Cancer type/model	Study design	Mechanism of action	References
PKM2	AP	CC	HCT116	↓ PKM2 expression	[111]
	PB2	HC	HCC-LM3, Bel-7402, SMMC-7721, Huh-7 and HepG2	↓ PKM2 expression	[112]
	EGCG	BC	T47D	↓ PKM2 expression ↓ PKM2 activity	[117]
	TAX, NEO, FIS, CG, EP	Only enzyme activity evaluated in vitro		↓ PKM2 activity	[118]
	QUE	BC	MCF-7, MDA-MB-231; BALB/c nude mice	↓ PKM2 level	[119]
		Colon mucosa (chemopreventive model) F344 rats		↓ PKM2 level	[120]
	SHI	BC, LC	MCF-7, MCF-7/Adr, MCF-7/Bcl-xL, MCF-7/Bcl-2, and A549	↓ PKM2 activity	[121]
		ME	B16-melanoma xenografts	↓ PKM2 level/activity	[122]
HK2	XA	CC	HCT116, HT29, SW620; HCT116, HT29 xenografts	↓ HK2 expression	[132]
	QUE	HC	SMMG-7721, BEL-7402 SMMC-7721 xenografts	↓ HK2 level	[134]
	EGCG	BC	T47D	↓ HK2 activity	[117]
	10v	CC	HCT116	↓ HK2 level	[136]
	GL-V9	BC	MCF-7, MDA-MB-231	↓ HK2 expression	[137]
	FV-429	BC	MDA-MB-231	↓ HK2 level/activity	[138]
	Gen-27	BC	MDA-MB-231	↓ HK2 expression	[139]
	ASG	HC	HepG2, Huh-7 H22, Huh-7 xenografts	↓ HK2 expression	[140]
LDH	MO	Only enzyme activity evaluated in vitro		↓ LDH enzymatic activity	[144]
	QUE	BC	MCF-7, MDA-MB-231	↓ LDH level	[119]
	LU-7OβD	Only enzyme activity evaluated in vitro		↓ LDH enzymatic activity	[145]
LDH, PFK	EGCG	BC	T47D	↓ LDH, PFK levels/activities	[117]
Aldolase, GAPDH,a-enolase	QUE	Colon mucosa (chemopreventive model) F344 rats		↓ aldolase, GAPDH, and a-enolase levels	[120]
GLUTs	AP, PH	PC	LNCaP, PC-3	↓ GLUT1/GLUT4 expression	[147]
	AP	PC	CD18, S2–013	↓ GLUT1 level	[148]
	HES	BC	MDA-MB-231	↓ GLUT1 level ↓ GLUT4 translocation	[151]
	KAE	BC	MCF-7	↓ GLUT1 level	[153]
	QUE, EGCG	BC	MCF-7, MDA-MB-231	↓ GLUT1 activity	[154]
	EGCG	BC	4T1	↓ GLUT1 expression	[117]
	QUE	BC	MCF-7, MDA-MB-231	↓ GLUT1 activity	[119]
	SI	Non-tumour CHO		↓ GLUT4 activity	[155]
HIF-1	BA	GC	AGS	↓ HIF-1 expression	[161]
	MF	BC	T47D	↓ HIF-1 activity	[162]
	OX-A	BC	MDA-MB-231	→ HIF-1 destabilisation	[163]
	EGCG	BC	T47D	↓ HIF-1 expression	[117]
	RES	LC, BC, CC	LLC, T47D, HT-29	↓ HIF-1 level	[164]

Explanatory notes: ↓ decrease, reduction, suppression; → induction

*PKM2*, pyruvate kinase muscle isoform 2; *HK2*, hexokinase 2; *LDH*, lactated hydrogenase; *PFK*, phosphofructokinase; *GAPDH*, glyceraldehyde-3-phosphate dehydrogenase, *GLUTs*, glucose transporters; *HIF-1*, hypoxia-inducible factor 1; *AP*, apigenin; *PB2*, proanthocyanidin B2; *EGCG*, epigallocatechin-3-gallate; *TAX*, taxifolin; *NEO*, neoeriocitrin; *FIS*, fisetin; *CG*, catechin gallate; *EP*, epicatechin; *QUE*, quercetin; *SHI*, shikonin; *XA*, xantohumol; *ASG*, astralangin; *MO*, morin; *LU-7OβD*, luteolin-7-O-β-D-glucoside; *PH*, phloretin; *HES*, hesperitin; *KAE*, kaempferol; *SI*, silibinin; *BA*, baicalein; *MF*, methylalpinumisoflavon; *OX-A*, oroxylin A; *RES*, resveratrol; *CC*, colon cancer; *HC*, hepatocellular cancer; *BC*, breast cancer; *LC*, lung cancer; *ME*, melanoma; *PC*, prostate cancer; *GC*, gastric cancer

## The Warburg effect from a clinical perspective: status quo

Investigating how anticancer therapies may target the Warburg effect has been of great interest since it was first described a century ago. During the last two decades, recent advances in this field have provided valuable knowledge regarding personalised detection and treatment of cancer. An example of its utilisation in cancer detection, treatment response and surveillance is positron emission tomography (PET-CT) imaging. As described by Croteau et al. [165] by assessing oxygen consumption and mechanisms of energy substrate consumption, through ^18^F-FDG uptake, this imaging modality has proven its great value for comprehensive management of cancers. After introduction of PET-CT imaging, innovative treatments have been developed to deliver more precise and directed radiotherapy fields, particularly in the setting of Hodgkin lymphoma. According to Girinsky et al. [166], historic radiation fields encompassed significant portions of healthy tissue, whereas modern radiation therapy techniques are able to limit radiation fields to focus on tumours identified by pre-treatment PET-CT, decreasing toxicities without compromising oncologic outcomes. For certain cancers, PET-CT is also utilised to evaluate treatment response after administration of chemotherapy [167]. This opens a new perspective for glucose-targeted drug therapies [168].

A parasitic effect, known as the ‘reverse Warburg effect’, by which tumour cells trigger a positive growth factor feedback from surrounding stromal cells after the ‘classical’ Warburg effect, was demonstrated in triple-negative breast cancer and is related to poor prognosis [169]. Based on these findings, clinical trials to assess the benefit of FDA-approved drugs such as metformin, hydroxychloroquine and *N*-acetyl-cysteine have been initiated due to their postulated property of blocking this particular pathway. Blocking a Warburg effect can be traced by PET-CT to assess treatment response [170]. Although an increased risk of aggressive BC specifically for young patients is well known amongst practitioners [171], the mechanisms for this phenomenon were unclear. The discovery made by Olga Golubnitschaja and colleagues described young patients with Flammer syndrome (FS) phenotype representing a specific group of risk predisposed to aggressive metastasing breast cancer, due to systemic vasoconstriction and tissue hypoperfusion [172–174] tightly linked to the systemic hypoxic–ischemic effects and Warburg transformation.

The Warburg effect also plays a role in novel methods for detection and treatment of prostate cancer. The employment of natural 1,4-naphtoquinone and its derivate 6-O-(1,4-naphtaquinone-2-yl)-D-glucose may improve selectivity in the Warburg effect blockade [175]. Further, clinical experiences with peroxisome proliferator-activated receptor ligands (PPAR ligands), which directly block the Warburg effect, yielded discordant results in terms of PSA control in 2 consecutive phase II trials [176, 177]. Although early development of prostate cancer in younger patients is recorded [178], current standard screening measures do not include patients under 45–50 years [179]. While strategies for early screening are currently under investigation, already available information points at metabolomics and radiomics to improve early diagnosis and personalised treatment opportunities [5]. The introduction of ^68^Ga prostate-specific membrane antigen PET-CT (PSMA-PET) and molecule dosage as the prostate cancer antigen 3 (PCA3), both with high selectivity for diagnosis and staging, has provided new options for individualised treatment planning, based on a patient’s risk profile [180–182]. Regarding metastatic disease, Radium-223 has a demonstrated effect on bony metastasis [183]. Its high selectivity for osteoblastic regions with high metabolic activity exemplifies how ‘anti-Warburg’ measures could play an advantageous role in targeted cancer treatment.

The ‘anti-Warburg’ approach, as described in this paper, is currently considered as being synergistic to comprehensive conventional therapies [184, 185]. Contextually, combined targeted therapies are strongly recommended for the cancer treatment setting.

## Individualised patient profiling as a powerful instrument for implementing targeted ‘anti-Warburg’ measures in the context of 3P medicine

Below, the most prominent examples are briefly summarised for the application of targeted ‘anti-Warburg’ measures categorised as three levels of prevention and according to patient stratification based on the individualised profiling. For more information, corresponding literature sources are provided.

### Targeted ‘anti-Warburg’ measures potentially effective for primary prevention in stratified groups of individuals with modifiable risk factors and reversible damage to health

A. Individuals demonstrating systemic hypoxic–ischemic lesions, due to disturbed microcirculation such as the following:

- Otherwise healthy individuals with Flammer syndrome phenotype who are typically affected by primary vascular dysregulation [186]; Flammer syndrome phenotype with predisposition to related pathologies can be diagnosed applying specialised questionnaires [172, 187, 188].
- Otherwise healthy individuals demonstrating disturbed microcirculation secondary to modifiable health condition such as low physical activity, due to sedentary lifestyle [189, 190], in overweight and obese individuals [191] who are per evidence at higher risk to develop cancer [192] compared to the general population, as well as in underweight individuals (malnutrition, dietary restrictions, anorexic condition), due to restricted energy resources, upregulation of HIF-1 [193] and potentially insufficient repair capacity [194].

B. Excessive stress exposure and accelerated ageing processes—both linked to preventable risk factors

Excessive stress exposure at an individual level might be linked to systemic vasoconstriction with consequent hypoxic–ischemic lesions and imbalanced damage-to-repair capacity that can be detected by ‘comet assay analysis’ [195, 196] and preventable accelerated ageing processes (e.g. in smokers and ageing population) [190]—all risk factors associated with cancer development and progression involving preventable contribution of Warburg effects [197].

C. Chronic inflammation and healing impairments

Chronic inflammation and healing impairments frequently resulting from disturbed microcirculation are well-acknowledged risks of cancer development and progression. Both chronic inflammation and healing impairment may occur to individuals in suboptimal health conditions [187, 194, 198]. Individualised patient profiling is the best instrument to detect cancer predisposition at a reversible stage of damage [199].

### Targeted ‘anti-Warburg’ measures potentially effective for secondary prevention in stratified patient groups with non-modifiable risk factors

A. Genetic predisposition to cancer

Genetic component plays a role in the Warburg effect as the central contributor to the cancer progression machinery [200]. Both inborn and acquired genetic alterations are involved in Warburg phenomenon influencing its severity [201]. Therefore, both genotyping and phenotyping are important for considering individualised ‘anti-Warburg’ treatment options.

B. Mitochondrial dysfunction

Although clarity is still needed, whether dysfunctional mitochondria are primary or secondary in Warburg effect [24], there is a consensus that mitochondrial dysfunction is central for cancerous transformation. Consequently, early detection of mitochondrial impairments is highly predictive for preventive ‘anti-Warburg’ treatments.

C. Benign tumours diagnosed

Benign tissue transformation is an acknowledged risk factor for a malignant development, for example, in breast cancer aetiology [202]. Considering benign tumours as potential cancer pre-stages, ‘anti-Warburg’ measures might be effective to prevent cancer development.

D. Clinically manifested relevant co-morbidities

As prominent examples, cardiovascular dysfunction and metabolic syndrome both linked to impaired circulation resulting in systemic hypoxic–ischemic lesions can be considered. To this end, patients with diabetic history are of increased cancer risk with poorer outcomes compared to non-diabetic patients [203]. Contextually, ‘anti-Warburg’ treatments have a potential to significantly improve individual outcomes in diabetic patients and patients with cardiovascular pathologies [204–207].

### Targeted ‘anti-Warburg’ measures potentially effective for tertiary prevention in stratified patient groups

A. Clinically manifested aggressive cancer subtypes with high metastatic potential

The incidence of aggressive breast cancer sub-types such as triple-negative metastasing tumours diagnosed in young females with normal and low BMI [6] is permanently increasing worldwide presenting a significant challenge for healthcare. Although their aetiology is still poorly understood, clear indications have been provided recently for the characteristic phenotype linked to the primary vascular dysregulation and some other signs and symptoms of Flammer syndrome [173, 174, 208]. The situation is particularly dramatic in the case of aggressive metastasing breast cancer, if diagnosed during pregnancy [209] (note: the publication was selected by Springer-Nature (2018) in the category ‘Medicine and Public Health’ as an article with a potential to change the world) [210]. In this group, an effective targeted chemoprevention adapted to the clinical situation is of great importance [211].

B. Palliative medical approach

Despite significant progress in early diagnostics and screening programmes during the last two decades, a large portion of malignancies are still diagnosed at advanced stages resulting in palliative approach only possible. This is particularly true for the liver malignancies, since almost all solid tumours are metastasing to the liver [212]. To this end, effective palliative approaches demonstrate a great capacity to turn liver malignancies into chronic health condition, if patient-tailored treatment algorithms are applied [213]. Contextually, ‘anti-Warburg’ measures utilising flavonoids and genoprotective strategies [196] might be helpful in stabilising health condition of patients undergoing selective internal radiation therapy (SIRT) or trans-arterial chemo-embolisation (TACE).

## Conclusion and expert recommendations

The rapid rise in cancer incidence and mortality is a global challenge [214]. Oncogenesis is a complex, multistage process including initiation, promotion and progression [215], as well as metabolic changes. It is well established that the increase of glucose uptake and lactate secretion due to metabolic changes in malignant cells constitutes the Warburg effect, an essential step of carcinogenesis [2]. Alterations in glucose metabolism associated with dramatically increased proliferation activity, progression and development of chemo-/radioresistance represent an important hallmark of malignancy [216]. Due to metabolic reprogramming, cancer cells exhibit increased levels of glucose uptake. This phenomenon is widely used for diagnostic and prognostic imaging of tumours using glucose analogues [217]. For many decades, PET demonstrates a non-invasive imaging method for evaluation of metabolic or functional changes in normal tissue as well as during disease conditions [218]. ^18^F-fluorodeoxyglucose (^18^F-FDG) is a glucose analogue that is widely used in combination with PET as a diagnostic tool for various types of cancer in clinical practice [219]. Consequently, FDG-PET is a clinically used technique for tumour imaging via increasing glucose uptake [220]. Further, magnetic resonance spectroscopy (MRS) utilising hyperpolarised ^13^C-pyruvate demonstrates great clinical utility, e.g. for prostate cancer patients. This technique is able to precisely delineate cancer relative to surrounding healthy tissue based on metabolic alteration in the cell [221]. Continuous progress in imaging techniques associated with specific cancer metabolism provides new opportunities for improving diagnostic procedures and individual patient outcomes [222].

Therapeutic strategies focused on specific cancer metabolism linked to the Warburg effect are currently under extensive consideration [174, 175]. To this end, naturally occurring bioactive compounds of plants demonstrate clinically relevant beneficial effects against cancer development and progression [223–230]. The combination use of phytochemicals and chemotherapeutical drugs may represent an optimal modality for anticancer therapy [231]. In this regard, the application of plant-derived natural substances as anticancer agents specifically against the Warburg phenotype is a promising strategy.

Flavonoids may modulate almost all key processes involved in carcinogenesis including apoptosis [232], proliferation [233], angiogenesis and metastatic progression [234, 235]. Their anticancer effects are directly associated with modulating energy and glucose metabolism, regulating specific enzymes of aerobic glycolysis and transporters required for glucose intake, which amongst others are involved in the Warburg effects characteristic for the cancerous cell transformation [111, 112, 117–122, 132, 134, 136–140, 144, 145, 147, 148, 151, 153–155, 161–164]. Still, in-depth analysis is essential to stratify individuals and patients for targeted application of flavonoids considering ‘anti-Warburg’ effects at the level of primary, secondary and tertiary prevention. To this end, individualised patient profiling is a powerful instrument for implementing targeted ‘anti-Warburg’ measures in the context of predictive, preventive and personalised medicine, which natural substances demonstrate a great potential to contribute to [236].

## List of abbreviations

1,3BGP: 1,3-bisphosphoglycerate
^18^F-FDG: ^18^F-fluorodeoxyglucose
2PG: 2-phosphoglycerate
3PG: 3-phosphoglycerate
ADP: adenosine diphosphate
AMPK: AMP-activated kinase
AP: apigenin
ASG: astralangin
ATP: adenosine triphosphate
BA: baicalein
BC: breast cancer
CC: colon cancer
CG: catechin gallate
DCs: dendritic cells
DHAP: dihydroxyacetone
EGCG: epigallocatechin-3-gallate
EP: epicatechin
F6P: fructose-6-phosphate
FBP: fructose-1,6-bisphosphate
FH: fumarate hydratase
FIS: fisetin
FOXO1: forkhead box subfamily O1
FS: Flammer syndrome
G3P: glyceraldehyde-3-phosphate
G6P: glucose-6-phosphate
GAPDH: glyceraldehyde-3-phosphate dehydrogenase
GC: gastric cancer
GE: genistein
GLUTs: glucose transporters
H2Bub1: monoubiquitinating of histone H2B
HC: hepatocellular cancer
HES: hesperitin
HIF-1: hypoxia-inducible factor-1
HK: hexokinase
HnRNPs: heterogeneous nuclear ribonucleoproteins
IL-1: interleukin 1
KAE: kaempferol
LBK1: liver kinase B1
LC: lung cancer
LLC: Lewis lung carcinoma
LDH: lactate dehydrogenase
LDHA: lactate dehydrogenase A
LU-7OβD: luteolin-7-O-β-D-glucoside
MAPK: mitogen-activated protein kinase
MCT: monocarboxylate transporters
ME: melanoma
MF: methylalpinumisoflavon
MMP-9: matrix metalloproteinase 9
MO: morin
MRS: magnetic resonance spectroscopy
NAD^+^: nicotinamide adenine dinucleotide (oxidised form)
NADH: nicotinamide adenine dinucleotide (reduced form)
NEO: neoeriocitrin
OX-A: oroxylin A
PB2: proanthocyanidin B2
PC: prostate cancer
PCA3: prostate cancer antigen 3
PEP: phosphoenolpyruvate
PET: positron emission tomography
PFK1: phosphofructokinase
PGI: phosphoglucose isomerase
PGK: phosphoglycerate kinase
PGM: phosphoglycerate mutase
PH: phloretin
PK: pyruvate kinase
PKM2: pyruvate kinase muscle isoform 2
PPAR ligands: peroxisome proliferator-activated receptor ligands
PPPM / 3PM: predictive, preventive, personalised medicine
PTB: polypyrimidine tract binding protein
pVHL: von Hippel–Lindau
QUE: quercetin
RES: resveratrol
ROS: reactive oxidative species
SCO2: synthesis of cytochrome C oxidase 2
SDH: succinate dehydrogenase
SHI: shikonin
SI: silibinin
SIRT: selective internal radiation therapy
SIX 1: sine oculis homeobox 1
TACE: trans-arterial chemo-embolisation
TAX: taxifolin
TCA: tricarboxylic acid cycle
TGF β: tumour growth factor β
TIGAR: TP53-induced glycolysis and apoptosis regulator
TPI: triosephosphate isomerase
VDAC: voltage-dependent anion-selective channel
VEGF: vascular endothelial growth factor
XA: xanthohumol
YY1: Yin Yang 1
